# Praziquantel for the treatment of schistosomiasis during human pregnancy

**DOI:** 10.2471/BLT.17.198879

**Published:** 2017-11-27

**Authors:** Jennifer F Friedman, Remigio M Olveda, Mark H Mirochnick, Amaya L Bustinduy, Alison M Elliott

**Affiliations:** a Center for International Health Research at Rhode Island Hospital, 55 Claverick Street, Suite 101, Providence, RI 02903, United States of America (USA).; b Department of Immunology, Research Institute for Tropical Medicine, Manila, Philippines.; c Division of Neonatology, Department of Pediatrics, Boston University School of Medicine, Boston, USA.; d Department of Clinical Research, London School of Hygiene and Tropical Medicine, London, England.; Correspondence to Jennifer F Friedman (email: Jennifer_Friedman@brown.edu).

## Abstract

In 2014, an estimated 40 million women of reproductive age were infected with *Schistosoma haematobium, S. japonicum* and/or *S. mansoni*. In both 2003 and 2006, the World Health Organization (WHO) recommended that all schistosome-infected pregnant and breastfeeding women be offered treatment, with praziquantel, either individually or during treatment campaigns. In 2006, WHO also stated the need for randomized controlled trials to assess the safety and efficacy of such treatment. Some countries have yet to follow the recommendation on treatment and many programme managers and pregnant women in other countries remain reluctant to follow the recommended approach. Since 2006, two randomized controlled trials on the use of praziquantel during pregnancy have been conducted: one against *S. mansoni* in Uganda and the other against *S. japonicum* in the Philippines. In these trials, praziquantel treatment of pregnant women had no significant effect on birth weight, appeared safe and caused minimal side-effects that were similar to those seen in treated non-pregnant subjects. Having summarized the encouraging data, on efficacy, pharmacokinetics and safety, from these two trials and reviewed the safety data from non-interventional human studies, we recommend that all countries include pregnant women in praziquantel treatment campaigns. We identify the barriers to the treatment of pregnant women, in countries that already include such women in individual treatments and mass drug administration campaigns, and discuss ways to address these barriers.

## Résumé

En 2014, on estimait que 40 millions de femmes en âge de procréer étaient infectées par *Schistosoma haematobium, S. japonicum* et/ou *S. mansoni*. En 2003 et 2006, l'Organisation mondiale de la Santé (OMS) a recommandé qu'un traitement au praziquantel soit offert, individuellement ou dans le cadre de campagnes de traitement, à toutes les femmes enceintes et allaitantes infectées par le schistosome. En 2006, l'OMS a également affirmé la nécessité d'essais contrôlés randomisés pour évaluer l'innocuité et l'efficacité de ce traitement. Néanmoins, certains pays ne suivent toujours pas la recommandation relative au traitement et dans d'autres pays, bon nombre de gestionnaires de programme et de femmes enceintes demeurent réticents à suivre l'approche recommandée. Depuis 2006, deux essais contrôlés randomisés sur l'utilisation du praziquantel au cours de la grossesse ont été menés: l'un sur *S. mansoni* en Ouganda et l'autre sur *S. japonicum* aux Philippines. Dans le cadre de ces essais, le traitement au praziquantel des femmes enceintes n'a pas eu d'effet notable sur le poids à la naissance, s'est révélé sans danger et a provoqué des effets secondaires minimes, similaires à ceux constatés chez les femmes traitées qui n'étaient pas enceintes. Ayant résumé les données encourageantes sur l'efficacité, la pharmacocinétique et l'innocuité tirées de ces deux essais et examiné les données de sécurité provenant d'études non interventionnelles sur l'homme, nous recommandons que tous les pays incluent les femmes enceintes dans des campagnes de traitement au praziquantel. Nous mettons en évidence les obstacles qui empêchent le traitement des femmes enceintes – dans des pays les incluant déjà dans des traitements individuels et des campagnes d’administration massive de médicaments – et décrivons des moyens permettant de surmonter ces obstacles.

## Resumen

En 2014, se estima que 40 millones de mujeres en edad reproductiva estaban infectadas con *Schistosoma haematobium, S. japonicum y/o S. mansoni.* Tanto en 2003 como en 2006, la Organización Mundial de la Salud (OMS) recomendó que todas las mujeres embarazadas y lactantes infectadas con esquistosoma recibieran tratamiento, con praziquantel, ya fuera individualmente o durante las campañas de tratamiento. En 2006, la OMS también informó de la necesidad de ensayos aleatorizados controlados para evaluar la seguridad y la eficacia de dicho tratamiento. Algunos países todavía tienen que seguir la recomendación sobre el tratamiento y muchos gestores de programas y mujeres embarazadas en otros países siguen siendo reacios a seguir el enfoque recomendado. Desde 2006, se han llevado a cabo dos ensayos aleatorizados controlados sobre el uso de praziquantel durante el embarazo: uno contra el *S. mansoni* en Uganda y el otro contra el *S. japonicum* en Filipinas. En estos ensayos, el tratamiento con praziquantel en mujeres embarazadas no tuvo un efecto significativo sobre el peso en el momento del nacimiento, pareció seguro y causó efectos secundarios mínimos, similares a los observados en sujetos no embarazadas tratadas. Después de resumir los alentadores datos sobre la eficacia, la farmacocinética y la seguridad de estos dos ensayos y revisar los datos de seguridad de los estudios observacionales en humanos, recomendamos que todos los países incluyan a mujeres embarazadas en las campañas de tratamiento con praziquantel. Identificamos las barreras para el tratamiento de mujeres embarazadas, en países que ya incluyen a mujeres en los tratamientos individuales y en las campañas masivas de administración de medicamentos, y analizamos las formas de abordar estas barreras.

## ملخص

شهد عام 2014 إصابة عدد من النساء في سن الإنجاب يُقدر بـ 40 مليون امرأة بعدوى البلهارسيا الدموية، و/أو البلهارسيا اليابانية، و/أو البلهارسيا المنسونية. وأوصت منظمة الصحة العالمية في كل من عام 2003 وعام 2006 بتقديم العلاج بدواء "البرازيكوانتيل" لجميع النساء الحوامل واللاتي يرضعن رضاعة طبيعية من المصابات بعدوى البلهارسيا، سواء كان تقديم العلاج على أساس فردي أو من خلال حملة علاجية. وصرحت أيضًا منظمة الصحة العالمية في عام 2006 بوجود حاجة لإجراء تجارب معشاة مضبطة بالشواهد لتقييم مدى فعالية هذا النوع من العلاج وتأثيره على سلامة المرضى. ولم تطبق بعض الدول حتى الآن التوصية بشأن هذا العلاج ولا يزال الكثير من مديري البرامج والنساء الحوامل في دول أخرى غير مقبلين على تطبيق النهج الموصى به. ومنذ عام 2006، تم إجراء اثنتين من التجارب المعشاة المضبطة بالشواهد بشأن استخدام دواء "البرازيكوانتيل" أثناء فترة الحمل: كانت إحداهما لمكافحة مرض البلهارسيا المنسونية في أوغندا، أما الأخرى فكانت لمكافحة مرض البلهارسيا اليابانية في الفلبين. ولم يظهر في هاتين التجربتين أي أثر ملحوظ على وزن الطفل عند الميلاد نتيجة لتلقي النساء الحوامل لعلاج "البرازيكوانتيل"، وبدا الدواء آمنًا ولم يسبب سوى أدنى حد من الآثار الجانبية التي تشبه تلك الآثار التي ظهرت لدى من تلقين العلاج من النساء غير الحوامل اللاتي خضعن للدراسة. وبعد تلخيص البيانات المشجعة المستمدة من هاتين الدراستين، فيما يتعلق بمدى فعالية العلاج ودراسة حركيات الدواء ومدى تأثيره على سلامة المرضى، وكذلك مراجعة بيانات السلامة المستمدة من الدراسات التي خضع لها البشر والتي لا تحتاج إلى إجراء تداخلي طبي، فقد توصلنا لتوصيات لجميع الدول بوجوب إدراج النساء الحوامل ضمن المستفيدين من حملات العلاج بدواء "البرازيكوانتيل". ونحن نعمل على تحديد العقبات التي تقف في سبيل علاج النساء الحوامل – في الدول التي تندرج فيها تلك النساء ضمن المستفيدين من العلاجات الفردية وحملات توزيع الدواء على نطاق جماهيري - وكذلك نناقش سبل مواجهة تلك العقبات.

## 摘要

2014 年，估计有 4000 万育龄妇女感染了埃及裂体吸虫、日本血吸虫和/或曼氏裂体吸虫。2003 年和 2006 年，世界卫生组织建议所有感染血吸虫的孕妇和哺乳期妇女都应在单独治疗或治疗活动中使用吡喹酮接受治疗。2006 年，世界卫生组织还表示需要进行随机对照试验，以评估这种治疗的安全性和有效性。一些国家还未按照建议进行治疗，另一些国家的项目管理人和孕妇仍然不愿遵循建议的治疗方法。自 2006 年以来，进行了两项关于在怀孕期间使用吡喹酮的随机对照试验：一项针对乌干达的曼氏裂体吸虫，另一项针对菲律宾的日本血吸虫。在这些试验中，孕妇使用吡喹酮治疗对婴儿出生体重没有显著影响，似乎很安全，产生了类似治疗未怀孕患者轻微的副作用。从这两项试验中总结了令人乐观的数据，涉及疗效、药物动力学和安全性，并评估了非介入性人类研究的安全性数据，我们建议所有国家的孕妇都应使用吡喹酮接受治疗。我们确定了孕妇治疗的障碍——在那些已经将孕妇纳入单独治疗和大规模药物管理活动中的国家——并讨论了解决这些障碍的方法。

## Резюме

В 2014 году около 40 миллионов женщин репродуктивного возраста были инфицированы *Schistosoma haematobium, S. japonicum* и/или *S. mansoni*. В 2003 и 2006 годах Всемирная организация здравоохранения (ВОЗ) рекомендовала назначать всем беременным и кормящим женщинам, инфицированным шистосомой, лечение с применением празиквантела как в индивидуальном порядке, так и во время массовых кампаний по лечению. В 2006 году ВОЗ также заявила о необходимости проведения рандомизированных контролируемых испытаний для оценки безопасности и эффективности такого лечения. Некоторые страны не последовали рекомендации по лечению, и многие руководители программ и беременные женщины в других странах по-прежнему неохотно придерживаются рекомендуемого подхода. С 2006 года было проведено два рандомизированных контролируемых исследования применения празиквантела во время беременности: одно в Уганде (в нем изучали применение этого препарата при инфицировании *S. mansoni*), другое на Филиппинах (при инфицировании *S. japonicum*). В этих исследованиях применение празиквантела у беременных женщин не оказывало существенного влияния на вес младенцев при рождении, оказалось безопасным и вызывало минимальные побочные эффекты, аналогичные тем, которые наблюдались при применении этого препарата у небеременных пациенток. Исходя из обнадеживающих данных по эффективности, фармакокинетике и безопасности, полученных в ходе этих двух исследований, и из пересмотренных данных по безопасности из неинтервенционных исследований, проведенных на людях, рекомендуется всем странам включать беременных женщин в кампании по лечению с применением празиквантела. Авторы выявляют препятствия для лечения беременных женщин в странах, которые уже включили таких женщин в массовые кампании по лечению с применением лекарственных препаратов или назначают им индивидуальное лечение, и обсуждают пути устранения этих препятствий.

## Introduction

In 2014, over 230 million individuals, including 40 million women of reproductive age, were estimated to be infected with *Schistosoma haematobium, S. japonicum* and/or *S. mansoni*.^1^ Despite the widespread availability of effective, praziquantel-based treatment, schistosomiasis remains the cause of substantial morbidity and mortality in many low- and middle-income countries.^2^ In a meta-analysis of the disability-related outcomes of endemic schistosomiasis, the disability weight assigned to schistosomiasis – which was based on the disease’s impact on a range of functional domains, ranged between 2% and 15%.^3^ The most recent Global Burden of Disease estimates, from 1980–2016, indicate that approximately 10 000 schistosomiasis-related deaths occur each year.^4^ Among non-pregnant subjects, schistosomiasis has been implicated as a contributor to undernutrition^5^^–^^11^, probably via the suppression of appetite^12^ and to inflammation-mediated cachexia.^13^ Schistosomiasis also contributes to the global burden of anaemia^7^^,^^9^^,^^14^^,^^15^ Individuals with heavy infections may lose so much blood in their stools and/or urine that they develop iron-deficiency anaemia.^16^^–^^19^ Pregnant women may experience any of these morbidities and others, e.g. hepatic fibrosis and the associated increased risk of oesophageal varices, at approximately the same rates as seen among non-pregnant individuals.^3^

Both *S. haematobium*^20^ and *S. mansoni*^21^ are known to contribute to the burden of anaemia in pregnancy, particularly at higher intensities of infection. The same may be true for *S. japonicum*. For example, in a recently completed randomized controlled trial in the Philippines, praziquantel given as a total dose of 60 mg per kg, to *S. japonicum*-infected pregnant women at 12–16 weeks’ gestation, led to improved maternal ferritin and a trend towards increased neonatal iron endowment.^22^

In 2000, urogenital schistosomiasis, which is largely caused by *S. haematobium*, was considered endemic in 53 countries in Africa and the Middle East.^23^ In areas where *S. haematobium* is endemic, women of reproductive age may experience chronic female genital schistosomiasis and this may place the women at increased risk of the acquisition and transmission of human immunodeficiency virus (HIV).^24^^,^^25^

Praziquantel was released in 1979, but the United States Food and Drug Administration (FDA) still places it in pregnancy class B^26^, indicating that animal reproduction studies^27^^,^^28^ have failed to demonstrate a risk to the fetus but no adequate and well-controlled studies in pregnant women have been conducted. In 2002, the World Health Organization (WHO) sponsored an informal consultation on the use of praziquantel during pregnancy and lactation. The report from that consultation, published in 2003, recommended that all schistosome-infected pregnant and breastfeeding women be considered high-risk groups and be offered treatment with praziquantel either individually or during treatment campaigns.^29^^–^^31^

Given the evidence of praziquantel’s safety in animal models, encouraging post-market surveillance data and its successful use in the treatment of neurocysticercosis during pregnancy, a similar recommendation was made in 2006, as part of WHO’s *Guidelines for Preventative Chemotherapy for Helminthiasi*s.^2^ These guidelines recommended that pregnant and breastfeeding women be included in mass administrations of praziquantel, but also noted the need for randomized controlled trials to assess praziquantel’s safety and efficacy when given to such women. Initially, the lack of sufficient safety data from controlled trials made many countries reluctant to follow this recommendation. Since 2006, however, there have been two relevant randomized controlled trials^22^^,^^32^ and the results of those trials have prompted some more countries, but not all, to follow the guidelines’ recommendations on praziquantel use.^33^ In Zanzibar, in the United Republic of Tanzania, where local guidelines do not recommend treatment of pregnant women, two of the most commonly cited reasons for not receiving praziquantel during community treatment campaigns were pregnancy and breastfeeding.^34^ China,^35^ Gabon,^36^ Kenya^37^ and Uganda^38^ have still to adopt policies to treat schistosome infections in pregnant women. Even in countries where the relevant national policy has been changed to include pregnant and breastfeeding women in mass administrations of praziquantel, some such women are still being excluded, because schistosomiasis programme managers and health-care providers remain unaware of the change. Millions of women of reproductive age may miss treatment for many years during repeated cycles of pregnancy and lactation.

Below, we review safety data from non-interventional human studies and summarize the data addressing safety, efficacy and pharmacokinetics from the relevant post-2006 randomized controlled trials.^22^^,^^32^ We identify current barriers to the treatment of pregnant women with praziquantel, in those countries where such treatment is already national policy and discuss ways in which these barriers may be overcome. Given the relevant data published since 2006, we recommend the inclusion of pregnant women in all praziquantel treatment campaigns.

## Praziquantel safety

### Non-interventional studies

Over more than 30 years of post-market experience with praziquantel, no reports of serious adverse events relevant to human pregnancy have been published, e.g. abortions, stillbirths or congenital anomalies. In a retrospective study in Sudan, 88 pregnancies in which there had been inadvertent praziquantel exposure, including 37 with exposure in the first trimester, were compared with 549 other pregnancies in which there had been no praziquantel exposure.^39^ None of the 88 exposed pregnancies ended in abortion or stillbirth and no congenital anomalies were noted by clinical examination of the babies born to the exposed women, or to the unexposed. In a small case series of travel-related acquisition of schistosomiasis, four pregnant women were treated with 60 mg praziquantel per kg, including two treated during the first trimester.^40^ Treatment did not culminate in any serious adverse events for the mothers or neonates. In a prospective study conducted in Sudan, 25 pregnant Sudanese women with *S. mansoni* infections were treated with a single dose of praziquantel, at 40 mg per kg, during different trimesters. Although one of these women experienced a spontaneous abortion at 10 weeks’ gestation and three weeks post-treatment, the frequency of abortion among the treated women was similar to the background rate in the study community.^41^ In the treatment of neurocysticercosis, pregnant women have been given daily doses of praziquantel for up to 21 days with no apparent adverse events.^42^

### Post-2006 trials

Two randomized, double-blind, placebo-controlled trials^22^^,^^32^ have been completed since WHO’s publication of its last, i.e. 2006, guidelines addressing the treatment of pregnant women with praziquantel.^2^

#### Uganda

In Uganda, where *S. mansoni* is endemic, women attending a hospital-based antenatal clinic were assigned into one of four treatment groups: placebo plus placebo, albendazole plus placebo, praziquantel plus placebo or albendazole plus praziquantel.^32^ As women who did not have schistosomiasis were included in the randomized sample, the study mimicked a typical mass drug administration in which infection status is not known at the time of treatment. Each single treatment was given during the second or third trimester, at a mean gestational age of 26.6 weeks. This large trial, which included 2507 women in its baseline analysis, found that praziquantel had no significant effect on maternal anaemia or birth weight, even among the women who were each confirmed to be infected with *S. mansoni* by the examination of a single stool sample. Perinatal mortality and congenital anomalies were as common in the placebo plus placebo group as in any of the other groups. Although, the infants born to mothers who had been given albendazole or praziquantel when pregnant, and infected with *S. mansoni,* showed a relatively high risk of infantile eczema,^43^ this did not translate into an increased risk of asthma in adolescence.^44^

#### Philippines

In Leyte in the Philippines, women who were infected with *S. japonicum* were recruited for another randomized controlled trial.^22^ Overall, 360 women were treated, at 12–16 weeks’ gestation, either with praziquantel, given as two doses, each of 30 mg per kg, with a four-hour interval, or with a placebo. Praziquantel treatment was found to have no significant impact on birth weight, i.e. the primary outcome, or on secondary outcomes such as the prevalences of low birth weight, intrauterine growth restriction and prematurity. Analysis of cytokine profiles indicated that such treatment also had no detectable effect on maternal inflammation, even by 32 weeks’ gestation. The treatment of schistosomiasis has complex effects on the immune system, including release of adult-worm antigens, enhanced responses to these antigens and a decline in immunoregulatory activity.^45^^,^^46^ At 32 weeks’ gestation, pregnant women have been found to have significantly higher blood concentrations of tumour necrosis factor-α when infected with *S. japonicum* than when uninfected, with mean values of 2.11 and 0.34 pg per mL, respectively.^47^ In the trial in Leyte, the corresponding concentrations, again at 32 weeks’ gestation, were 1.6 pg per mL for the placebo group and 1.3 pg per mL for the praziquantel group.^22^ These results indicate that, in the trial, praziquantel treatment did not resolve inflammation in the ensuing five or six months. The lack of a significant impact of praziquantel on birth weight may also be a reflection of the generally light infections; only 9.7% of the women enrolled in the trial had moderate or intense *S. japonicum* infections.

In the trial in Leyte, all of the pregnant women given praziquantel showed parasitological cure at 22 weeks’ gestation.^22^ Praziquantel treatment culminated in increased maternal serum ferritin levels at 32 weeks’ gestation as well as a trend towards improved neonatal iron endowment. Such treatment was not found to have any significant impact on maternal haemoglobin or anaemia risk during the trial. However, significant improvements in haemoglobin may take more than three months to show after praziquantel treatment^5^ and the low prevalence of maternal anaemia and the generally low intensity of the *S. japonicum* infections in the trial may have masked any potentially beneficial effects of praziquantel on maternal anaemia. Praziquantel treatment was well tolerated in the trial and the reactogenicity rates in the treated pregnant women were similar to those observed in treated non-pregnant participants. Importantly, such treatment had no significant effect on key safety outcomes such as abortion, congenital anomalies and intrauterine fetal death.^48^

### Pharmacokinetics

Praziquantel pharmacokinetics in non-pregnant adults are characterized by peak concentrations one to three hours after a dose and a terminal half-life that ranges between 0.8 and 1.5 hours. During pregnancy, however, important physiological changes could affect drug pharmacokinetics. For example, there is a decrease in gastric emptying, decreased bioavailability, decreased drug binding, altered liver metabolism and increased elimination.^49^ The pregnancy-related changes that occur in pharmacokinetic distribution and exposure parameters include decreased peak plasma concentrations, longer elimination half-lives and a reduction in the so-called area under the curve, which leads to lower levels of drug exposure.^50^ The appropriate drug dose and regimen to be given during gestation must therefore be carefully determined.

The first detailed study of the pharmacokinetics of praziquantel in pregnant women was conducted as part of the randomized controlled trial in Leyte. The results of this study, which included assays quantifying the concentrations of praziquantel in the milk of treated women have yet to be published. A preliminary analysis indicated that the pharmacokinetics of praziquantel in women in early pregnancy, women in late pregnancy and postpartum subjects were similar (M Mirochnick, Department of Pediatrics, Boston University School of Medicine, unpublished data, 2017). For example, the corresponding mean areas under the curve were found to be 8.9 mg-hour per L for women in early pregnancy, 15.0 for women in late pregnancy and 13.1 for postpartum subjects. Drawing conclusions from such data are difficult as there has only been one previous study of the pharmacokinetics of praziquantel in a study area with endemic *S. japonicu*m, and that was confined to just two non-pregnant women.^51^ The results of the ongoing pharmacodynamic analysis from the Leyte trial, will hopefully help clarify the optimal dosing of praziquantel during pregnancy.

## Policy implications

Since 2006, when WHO last published a recommendation on the use of praziquantel in pregnant and breastfeeding women, considerable relevant information has been published or, at least, analysed. This information, from two randomized controlled trials and additional observational and pharmacokinetic studies, provides support for WHO’s recommendation. In the trials, no increased risk of adverse pregnancy or neonatal outcomes was observed when praziquantel was given either at 40 mg per kg during the second or third trimester or at a total dose of 60 mg per kg at 12–16 weeks’ gestation. Given this improved evidence of the drug’s safety, we recommend that pregnant and breastfeeding women now be included in all praziquantel-based mass drug administrations.

Policies for the treatment of pregnant women with praziquantel must consider the timing of such treatment. The safety of giving praziquantel to pregnant women during the first trimester has not been well assessed. However, the apparent lack of teratogenic effects in mice, rats or rabbits given praziquantel while pregnant indicates that the drug may be safe to use during the classic teratogenic period of the human first trimester.^28^^,^^52^ The observations made, retrospectively, on women inadvertently exposed to praziquantel during the first trimester,^39^ case reports of treatment during the first trimester with no untoward effects^40^ and the encouraging results of over 30 years of post-market surveillance involving many millions of doses provide additional reassurance. Given the lack of data suggesting that praziquantel poses a risk to mothers or fetuses and the costs and logistical barriers of pregnancy testing, we do not advocate that routine pregnancy testing of women of reproductive age forms a part of any campaign of praziquantel-based mass drug administrations.

The possibility of treating pregnant women outside of mass drug administrations needs to be considered. Based on the demonstrated safety of praziquantel during the second and third trimesters, we recommend the treatment of pregnant women during routine visits for antenatal care if the women request such treatment or show the symptoms of schistosomiasis. We refrain from recommending that all pregnant women in areas with endemic schistosomiasis be treated with praziquantel as part of a routine package of antenatal care, at least until there has been a more detailed analysis of the potential costs and benefits. In the Ugandan randomized controlled trial, such an approach, in which all women were treated regardless of their infection status, did not reduce maternal anaemia status or increase birth weights significantly.^32^

Finally, provision of praziquantel for non-pregnant women of reproductive age, either as an individualized treatment or part of a mass drug administration, is important for women’s own health and well-being. If opportunities for such treatment are missed, women are placed at increased risk of chronic morbidity and the intense schistosome infections that are particularly associated with anaemia and undernutrition.^1^^,^^3^^,^^5^^,^^6^^,^^14^^,^^15^ If left untreated, women living in areas endemic for *S. haematobium* face developing female genital schistosomiasis which may, in turn, increase the risks of acquiring and transmitting HIV.^25^^,^^53^

## Barriers to policy adoption

Although recent progress in many relevant areas should increase the access of pregnant women to praziquantel treatment, barriers to adopting polices for such treatment still remain. The relevant information collected since 2006 should provide reassurance to countries that have not yet adopted WHO’s recommendation on the treatment of pregnant and breastfeeding women with the drug. By creating this update, we hope to mitigate any remaining concerns of policy-makers. 

Since the expiration of Bayer’s patent in 1994, the price of praziquantel, which was once a substantial barrier to the drug’s widespread use, has plummeted, to approximately 0.08 United States dollar (US$) per 600-mg tablet or approximately US$ 0.20–0.30 per treatment course in 2017.^54^ Furthermore, in recent years, the supply of praziquantel has increased dramatically. In 2016, to meet the need for treatment, Merck Soreno pledged to donate 250 million tablets of praziquantel annually.^55^ Praziquantel is also now available from the United Kingdom of Great Britain and Northern Ireland’s Department for International Development, the United States Agency for International Development, the World Bank and World Vision, such that sufficient supply should be available to provide close to 130 million treatments annually.^55^

Finally, even among countries that have adopted policies to include pregnant and breastfeeding women in praziquantel-based mass drug administrations, substantial gaps still exist in the implementation of those policies. One issue is the prioritization of school-based or community-based mass drug administrations in some areas. School-based programmes risk missing most women of reproductive age, as well as adolescent girls who cannot afford to attend school, especially if the schools involved are primary. Many programmes for the support of mass drug administrations are so poorly staffed and resourced that treatments cannot be delivered at the intervals indicated by the community prevalence of schistosome infection. Community health workers need to be resourced better and educated on the safety of praziquantel for pregnant and breastfeeding women.

In a collaboration with regulatory staff at the FDA and United States  National Institutes of Health, we began work to change the Food and Drug Administration’s pregnancy designation for praziquantel, from class B to class A. In 2015, however, FDA moved away from the letter categories for pregnancy designations, to a more narrative approach.^56^

## Conclusion

Based on recent evidence of the safety of praziquantel in human pregnancy, we recommend that, in areas with endemic schistosomiasis, women of reproductive age, including pregnant and breastfeeding women, be treated with praziquantel either individually, in local antenatal clinics, or in mass drug administrations. As more women are treated, surveillance to identify any untoward effects of treatment should be continued. It is expected that treatment may improve both maternal and neonatal iron stores.^22^ Surprisingly, two randomized controlled trials did not reveal an impact of the treatment of pregnant women with praziquantel on subsequent birth weights. According to data from animal models and observational and mechanistic studies on humans, however, it remains possible that, although schistosomiasis adversely affects pregnancy outcomes, treatment during gestation is too late to impact birth outcomes during that pregnancy. Individualized treatment during antenatal care and treatment in mass drug administrations may, however, increase the likelihood that women enter subsequent pregnancies free of infection. Regular treatment is likely to impact both the overall prevalence of infection and the prevalence of the intense infections that are most closely associated with maternal morbidity.
